# Benefits of high-magnification and color information in computational pathology—An exploratory review

**DOI:** 10.1016/j.jpi.2026.100701

**Published:** 2026-07-30

**Authors:** Nico Haisch, Friederike Liesche-Starnecker, Gregor Grambow, Daniel Hieber

**Affiliations:** a Dept. of Computer Science, Aalen University, Beethovenstraße 1, Aalen 73430, Baden-Württemberg, Germany; b Institute of Neuropathology, Ulm University Medical Center, Faculty of Medicine, Ulm University, Albert-Einstein-Allee 23, Ulm 89081, Baden-Württemberg, Germany

**Keywords:** Computational pathology, Deep learning, High-resolution, Color information, Whole-slide imaging, Magnification, Literature review

## Abstract

**Introduction:**

The analysis of gigapixel high-resolution whole-slide images (WSIs) with rich color information using deep learning (DL) models is a routine task in computational pathology (CPath). However, the necessity of such information-rich inputs remains insufficiently explored.

**Objectives:**

This literature study aims to collect evidence for the benefit, or its absence, of using WSIs with high magnification levels and full color information in CPath DL models.

**Methods:**

Due to the scarcity of domain-specific studies, the exploratory review includes research from related domains, such as broader medical imaging and computer vision in general. The search was conducted using different permutations of keywords in established databases such as IEEE Xplore and PubMed. The retrieved literature was pre-screened by a researcher and relevant work was screened by two researchers independently.

**Results:**

The analyzed literature reinforces the lack of direct empirical evidence for using high magnification WSIs with full color information in CPath. Conversely, some findings suggest that reducing image resolution can often maintain high DL accuracy for many classification and detection tasks. Similarly, thoughtful reduction of color information, such as advanced grayscale conversions, can preserve critical diagnostic features with minimal impact on DL performance.

**Conclusion:**

The review underscores the need to critically re-examine prevailing conventions on WSI magnification and color usage to optimize DL-driven CPath applications. The lack of direct evidence warrants further research and the establishment of reliable benchmarks in this domain.

## Introduction

Computational pathology (CPath) has emerged as a key application area for deep learning (DL)-based medical image analysis. The digitization of histological slides into whole-slide images (WSIs) enables large-scale computational analysis of tissue morphology and has opened new opportunities for automated diagnosis, prognosis, and biomarker discovery.^1^, ^2^, ^3^, ^4^ Consequently, CPath has rapidly adopted methodological advances from computer vision (CV) and medical imaging, particularly from radiology, where DL has been successfully applied for over a decade.^5^, ^6^ Beyond medical imaging, domains such as geospatial image analysis share key technical characteristics with CPath, including gigapixel-scale images, patch-wise processing, and multi-channel data representations, making them a relevant source of methodological insights.^7^

However, this close methodological inheritance has also led to largely unquestioned design choices. Despite the differences in image characteristics and use cases, most CPath pipelines default to using the highest available image magnification and full color information.^1^, ^8^, ^9^ Whereas this approach has yielded impressive results across a wide range of tasks, including tissue segmentation, tumor classification, and outcome prediction, the optimality of these choices has rarely been examined. Images acquired at maximum magnification impose substantial computational, storage, and energy costs, and can complicate reproducibility, data sharing, and deployment in clinical and multi-center settings.^1^ Especially developing countries often lack the resources for the required large DL models.^10^, ^11^ At the same time, it remains unclear whether all downstream tasks truly benefit from maximum spatial detail and full color fidelity, or whether comparable performance could be achieved using reduced magnification or simplified color representations.

The use of maximum magnification not only contrasts with the viewing behavior of pathologists, who spend the majority of time on lower magnifications,^12^ but also contrasts other areas of CV. Investigations demonstrated that the effects of input resolution, resizing strategies, and color space transformations using aggressive downsampling or reduced color information can preserve or even improve task performance while reducing computational demands in certain settings.^13^, ^14^, ^15^, ^16^ However, systematic analyses of the effects of magnification and color information are largely missing in CPath, where high-magnification and color-rich inputs are often treated as inherently beneficial rather than as tunable design parameters.

The objective of this exploratory literature review is to examine existing evidence on the role of image magnification and color information in CPath. Where pathology-specific evidence is scarce, relevant findings from radiology and general CV are considered to contextualize current practices and highlight transferable insights.

By mapping the current state of the literature, this review seeks to encourage a more critical and principled consideration of magnification and color in CPath, moving beyond inherited conventions toward task-aware, efficient, and reproducible methodological choices.

## Methods

An exploratory review methodology was employed to examine the current evidence regarding the impact of magnification and color information on DL performance in CPath. Specifically, the review addresses the following questions:

1. How does image magnification/spatial resolution affect DL performance in CPath?
2. What impact does color information have on DL model performance in CPath?

Given the limited domain-specific literature, the scope was extended to include relevant studies from geospatial imaging, medical imaging, and conventional CV.

### Conceptual clarification

In CPath, “resolution” and “magnification” are frequently used interchangeably, although they refer to distinct concepts. In conventional CV, resolution typically denotes the pixel dimensions of the model input (e.g., 224 × 224 or 512 × 512 pixels), obtained via resizing of a pre-acquired image. In radiology, high-resolution often refers to input sizes of 512 × 512 pixels or larger.^17^

In contrast, WSIs are acquired at predefined optical magnifications (commonly 20× or 40×), which determine the physical sampling resolution of tissue (e.g., μm/px). A fixed-size patch (e.g., 512 × 512 pixels) extracted at 40× represents a substantially smaller physical tissue area than the same patch extracted at 10×. Consequently, reducing magnification does not change the resolution of the image but represents different physical tissue areas and levels of morphological detail.

### Search strategy

The literature search was conducted across PubMed, IEEE Xplore, ACM Digital Library, and Google Scholar, covering a wide range of medical and computer engineering research. The search used combinations of the terms “computational pathology” OR “digital pathology,” “resolution,” “downsampling,” “color information,” “grayscale,” and “deep learning” OR “machine learning” OR “computer vision” OR “image processing.” Due to the large heterogeneity and low availability of CPath-specific research, no generic query could be created and an exploratory search with permutations of the keywords was employed instead of a systematic procedure. Literature published up to June 28, 2025 was included.

This approach provided a more comprehensive understanding of the topic by drawing insights from related fields where direct evidence in CPath is unavailable or limited.

The search itself did not follow a PRISMA approach. Records were initially screened based on titles and abstracts by a researcher with a computer science background. The selected full texts were then independently screened by a computer science and a medical informatics researcher for final inclusion and knowledge extraction.

### Selection and inclusion criteria

The literature selection process followed clearly defined inclusion and exclusion criteria. The inclusion criteria were: (1) Peer-reviewed journal articles and conference papers that explicitly examine the effects of image resolution on the performance of DL models in CPath or general CV contexts. (2) Studies that investigated the role of color information in CPath or general CV contexts. (3) Research addressing computational efficiency, storage considerations, or processing speed related to high-resolution color images.

Exclusion criteria were applied to maintain focus and quality standards. These excluded: (1) Articles without empirical evidence. (2) Studies focusing solely on traditional image processing techniques without ML components. (3) Research with insufficient technical details regarding resolution or color space specifications. (4) Studies focusing solely on stain normalization, as the benefits of normalization techniques have been sufficiently validated.^18^, ^19^

## Results

In the following, findings from the review on high-resolution and color information are reported in two structured subsections. Within each subsection, all identified studies are presented in an overview table, ordered by their relevance to CPath applications. As anticipated during the development of the methodology, the limited amount of CPath research addressing these fundamental questions necessitated the incorporation of evidence from related fields to offer a thorough analysis of the existing knowledge.

Although geospatial imaging differs from CPath in its end-user context, it shares several technical characteristics, including large-scale high-resolution imagery, patch-wise processing, and multi-channel data representations. Therefore, geospatial imaging studies were ranked above general CV studies in terms of relevance to CPath. However, evidence from non-CPath domains should generally be treated as indirect evidence that can support interpretation and hypothesis generation, rather than definitive evidence for CPath-specific recommendations.

19 studies were considered relevant: 9 addressed magnification/resolution, 11 addressed color-related aspects, with one study addressing both.

### Effect of high magnification

The effect of image magnification on DL performance has been investigated across both computational pathology and related computer vision domains. Whereas high magnification is commonly assumed to be beneficial, existing studies suggest a more nuanced picture, where task-specific trade-offs between spatial detail, contextual information, and computational efficiency play a critical role. In particular, lower magnifications can retain sufficient discriminative information for certain tasks while significantly reducing computational demands.

Table 1 summarizes representative studies and highlights how the relevance of magnification varies across domains and application scenarios. An expanded version of the table is available in Supplementary Table S1.

**Table 1:** Overview of papers handling the effect of high magnification/resolution in CV. The domain either is focused on *CPath*, medical imaging *MI*, or geospatial imaging *GSI*. The tasks are divided into segmentation *SEG*, classification *CLS*, and object detection *OD*.

Paper	Domain	Task	Conducted experiment
Neary-Zajiczek et al.^20^	CPath	CLS	Histopathological breast tissue classification on different magnifications.
Wiebe et al.^21^	CPath	CLS	Histopathological lung tissue classification on different magnifications.
Peng et al.^22^	CPath	OD	Detection of morphological structures in histopathological tissue.
Ardohain and Fei^23^	GSI	SEG	Landscape segmentation in satellite images with different magnifications.
Yang and Hu^24^	GSI	CLS	Crop classification at different resolutions.
Sabottke et al.^25^	MI	CLS	X-ray classification on different resolutions.
Rajaraman et al.^26^	MI	SEG	X-ray segmentation of tuberculosis lesions on different resolutions.
Hirahara et al.^27^	MI	CLS	X-ray classification on different resolutions.
Wollek et al.^28^	MI	CLS	X-ray image classification on different resolutions.

#### Evidence from computational pathology

DL models showed substantial tolerance to reduced magnification for classification tasks on multiple datasets for both breast^20^ and lung histopathology.^21^

Neary-Zajiczek et al.^20^ investigated breast histopathology classification using three datasets (BACH,^29^ BreaKHis,^30^ and PatchCamelyon^31^) and simulated reduced optical resolution (4× magnification up to 40× magnification) by lowering numerical aperture (NA). Their results demonstrate that strong performance can be maintained under substantial resolution degradation, particularly when models are retrained on degraded images. For example, models trained on BreaKHis images degraded to simulate NA = 0.04 maintained an accuracy of around 97% in retrained models (compared with 99% at maximum resolution). In contrast, applying degradation at inference without retraining caused more pronounced performance drops, especially at higher degradation levels and for some datasets, indicating that robustness is not automatic but depends on alignment between training and deployment resolution.

A convergent pattern is reported by Wiebe et al.,^21^ who examined spatial resolution effects on lung histopathology classification (TCGA lung cancer; >2000 slides^32^, ^33^) using tiled inputs degraded via low-pass filtering to resolutions from 8 to 128 μm/px. In contrast to the previous study, their starting resolution was at 2.5× (4 μm/px) rather than 40×. While performance decreased with decreasing spatial resolution, robust discrimination (AUC ≥ 0.95 across classes on the validation set) remained possible at 4–16 μm/px. Below this range, discrimination between LUAD and LUSC deteriorated, suggesting that subtype-specific morphology requires finer spatial detail than coarse normal-vs-tumor separation. Importantly, normal tissue remained distinguishable from cancer at much coarser resolution (up to 128 μm/px), consistent with the notion that “diagnostic scale” is task dependent.

Peng et al.^22^ systematically tested downsampling and compression effects on glomeruli detection in kidney WSIs scanned at 40×. Across multiple object detection architectures (e.g., Faster R-CNN, Mask R-CNN), downsampling by 8× produced the best overall results while also increasing computational efficiency and significantly reducing file sizes. Moderate JPEG compression (e.g., 40%) further reduced storage burden (reported >6000× overall) with negligible accuracy loss (mAP ≈ 0.781–0.780). Notably, glomeruli are large structures that were not fully visible at 40× or 4× downsampling. 8× downsampling was able to perfectly capture most glomeruli as whole structures. This again illustrates a task-specific optimum rather than a generalizable rule.

#### Evidence from analogous computer vision domains

Geospatial imaging studies mirror CPath's scaling constraints (large, high-resolution, multi-channel imagery) and offer structured evidence on resolution trade-offs.

Ardohain and Fei^23^ trained 36 segmentation models using high-resolution (3DEP, 1.5 m) vs. low-resolution (NLCD, 30 m) training labels across WV2 (1.84 m), Sentinel-2 (10 m), and Landsat-8 (30 m) imagery, evaluated on an independent manually interpreted test set digitized from 0.3 m ArcGIS basemap imagery. Whereas 3DEP labels and WV2 data consistently yielded the best performance, the maximum F1 gain over NLCD was only 0.027 on WV2 imagery, indicating diminishing returns of high-resolution annotation when imagery is already high-resolution. As WV2 image data consistently yielded the best results, this supports the benefit of high magnification in this study.

Yang and Hu^24^ studied crop classification while varying both spatial and spectral resolution (hyperspectral UAV data). They reported that extremely high spatial resolution (0.043 m) did not necessarily improve discrimination at the relevant scale, and that reducing spatial resolution to an intermediate range (0.5–1 m) maintained accuracy. Degradation became harmful mainly when resolution was reduced beyond a task-relevant threshold (e.g., overly aggressive downsampling). This again supports the presence of intermediate optima and diminishing returns at very high-resolution.

#### Evidence from medical imaging

Across radiology studies (e.g., chest X-ray classification and segmentation), a consistent pattern emerges: performance improves with increasing input resolution up to a point, then plateaus or shows only marginal gains, with task and architecture determining the exact optimum. These peaks are generally in the mid range of the tested resolutions,^25^, ^26^ with one study where even low resolutions are superior (64 px out of the tested range 32–1024 px).^27^ Another study reported the highest AUC at maximum tested resolution, however, with small absolute improvements (≈1.5% AUC gain between 256 px and 1024 px resolution).^28^ Collectively, these results reinforce that “maximum resolution” is not a universally optimal default and must be justified relative to the downstream objective (classification vs. small-lesion localization vs. segmentation) and available data.

### Effect of color information

The role of color information in DL models for image analysis has likewise been explored in both computational pathology and broader computer vision research. Although full color is typically treated as a default input representation, several studies indicate that reduced or transformed color spaces can preserve relevant information and, in some cases, even improve robustness or generalization.

Table 2 provides an overview of relevant studies and situates them with respect to their domain of origin. An expanded version of the table is available in Supplementary Table S2.

**Table 2:** Overview of papers addressing the effect of color information in CV. The domain either is focused on *CPath*, geospatial imaging *GSI*, micrograph imaging *MGI*, or general *CV*. The tasks are divided into segmentation *SEG*, classification *CLS*, object detection *OD*, and regression *REG*.

Paper	Domain	Task	Conducted experiment
Srisermphoak et al.^34^	CPath	CLS + SEG	Evaluated effect of novel grayscale conversion algorithm vs. color images for CPath.
Dias et al.^35^	GSI	SEG	Comparing grayscale and color images for semantic segmentation in aerial imaging.
Yang and Hu^24^	GSI	CLS	Crop classification on different numbers of spectral bands.
Pei et al.^36^	MGI	CLS + REG	Comparison of model performance on grayscale and colored micrograph images.
Buhrmester et al.^37^	CV	CLS	Comparison of classification performance on different types of color representation including grayscale and default RGB.
De and Pedersen^38^	CV	CLS	Comparison of classification performance on different types of color representation including grayscale and default RGB.
De^39^	CV	SEG	Comparison of segmentation performance on different types of color representation including grayscale and default RGB.
Bui et al.^40^	CV	CLS	Comparison of classification performance of grayscale and RGB with custom models.
Bhatta et al.^41^, ^42^	CV	CLS	Comparison of grayscale vs RGB model for face recognition.
Al-Mohana et al.^43^	CV	CLS	Classification of blood smear images with grayscale and RGB.

#### Evidence from computational pathology

Direct evidence on color reduction in CPath is extremely limited: only one study was identified that systematically compares full color information and reduced-color representations for histopathology.

Srisermphoak et al.^34^ introduced an attention-inspired grayscale conversion method (ACSRM) designed to encode inter-channel relationships before producing a single-channel representation. The method was evaluated on the PANDA,^44^ CAMELYON16,^31^ and OES^45^ datasets. Both segmentation and classification tasks were evaluated. ACSRM reduced file size by approximately 66% while yielding performance comparable to, and in several settings slightly superior to, RGB baselines for CNN-based segmentation models. RGB models only produced superior results on the OES dataset. As a baseline standard, luminance-based grayscaling was also evaluated, but produced inferior results.

This study challenges the implicit assumption that full color information is essential for high-accuracy histopathological DL. However, the use of simple grayscale methods alone is not sufficient to generate comparable results and dedicated conversion algorithms may be required.

Notably, the benefit of ACSRM does not translate to transformer models.

#### Evidence from analogous computer vision domains

Dias et al.^35^ evaluated full color information vs. grayscale semantic segmentation on two high-resolution aerial datasets using a W-Net architecture. Color provided a small but consistent advantage (difference in weighted F1 ≈+1.4% and ≈+1.0% on both datasets). Class-level differences were generally small, indicating that well-trained models can compensate for missing chromatic information in complex segmentation tasks, albeit with a slight performance penalty.

Pei et al.^36^ investigated color vs. grayscale inputs and preprocessing choices on micrographs of Mg—Li alloy. They report that full color information does not significantly improve CNN performance over grayscale in their micrograph classification use case. Instead, the emphasis is on preprocessing choices (e.g., center cropping and normalization) for performance maximization.

Yang and Hu^24^ also researched the effect of spectral band reduction in aerial imaging. Whereas spectral bands are not directly translatable to color channels such as RGB, this provides indirect insight into the potential need for different input channels. Their results show that reducing the number of spectral channels typically causes only slight accuracy decreases based on the selected model architecture. They conclude that using all available spectral bands is not necessary for reliable classification and that computational costs can be reduced by selecting an appropriate subset of channels.

#### Evidence from other computer vision domains

Further studies indicate that color importance depends strongly on dataset characteristics and label semantics. Buhrmester et al.^37^ tested multiple color representations and grayscale across datasets ranging from object recognition to landscape and person detection tasks. When models were trained on RGB but evaluated on grayscale, the error rate increased by ≈8% on CIFAR-10 (suggesting color contributes meaningfully), whereas in person/background tasks, removing color had little effect. For color-dependent datasets (e.g., FlickrScene, CIFAR-100), performance drops were larger, particularly for classes defined primarily by color.

Across other CV domains, the benefit of color is highly dataset- and task-dependent. Cross-condition tests show that training models on color images, but conducting inference on grayscale can be negligible for shape/texture-driven problems (e.g., PersonFinder) but substantially harmful for more color-semantic datasets (e.g., CIFAR/FlickrScene).^37^ Further research discovered that both grayscale and RGB-based face detection models yield similar accuracy and similar early layer features making them interchangeable for face classification.^41^ Other large-scale benchmarks that systematically remove channels or saturation reported consistent accuracy drops for classification^38^ and segmentation under grayscale/distorted color spaces across many CNNs and transformer models.^39^ Designing models specifically for grayscale input, rather than simply reusing RGB-oriented architectures, can improve accuracy and efficiency. A k-means filterbank CNN–RNN with grayscale input was able to outperform RGB-based models for object detection.^40^ The use of simple grayscale transformation without model changes of malaria blood-smear images caused only a minor performance reduction, highlighting that color can be redundant when discriminative cues are predominantly structural.^43^

## Discussion

Overall, the results of this exploratory literature review show a significant lack of evidence-based research regarding foundational design decisions in CPath investigating the effect of magnification and color information on DL model performance.

However, based on the three available CPath studies evaluating the effect of magnification on model performance, the evidence suggests that magnification can be reduced while retaining high performance for classification and object detection tasks across multiple datasets and model architectures (see Section "Effect of high magnification"). Whereas no general magnification recommendation can be made, different task-specific optimal values were discovered. These findings align with studies in radiology and geospatial imaging, where diminishing returns beyond intermediate resolution and cost–benefit trade-offs have been more thoroughly explored.

Rather than acquiring and processing WSIs at maximum magnification by default, magnification could be treated as a task-specific hyperparameter optimized relative to the downstream endpoint (e.g., tumor presence vs. subtype vs. cell-level grading vs. structure detection). Whereas more studies adjust magnification to their use-case and work with reduced magnification, only few studies evaluate the effect of different magnifications explicitly.^20^, ^21^, ^46^

The effect of color information and its reduction in CPath is currently anchored by a single empirical study that demonstrates that thoughtfully engineered grayscale representations can preserve or even improve performance for CNN-based models while reducing storage and transmission burden.^34^

Contrary to this, findings from aerial imaging and general CV reinforce that color often provides a modest advantage, with the magnitude of the benefit depending on label semantics and the extent to which discriminative cues are chromatic rather than structural. Nevertheless, depending on the task, grayscale-based models can prove superior with lower computational overhead and storage requirements.

As a side finding, the importance of preprocessing and custom architectures for grayscale images was recognized. The use of appropriate preprocessing steps often provides superior performance boosts compared to the simple use of color information. Moreover, specifically engineering ML models to work with grayscale rather than reusing models conceptualized for color input proved advantageous.

The current study comes with some methodological and domain-inherent limitations. Due to the limited evidence-based research on the topic and heterogeneity of results, an exploratory review approach was employed rather than a systematic one. While this limits the reproducibility, it allowed for a more flexible literature search and result aggregation, fitting for a first analysis of this topic. However, at the same time, this could have resulted in relevant papers being missed in the review process.

For magnification research, no studies were discovered evaluating the effect of magnification on CPath segmentation tasks, limiting the findings to classification and object detection. Whereas findings from other domains can be used to estimate the effect, no clear evidence is available.

Additionally, segmentation metrics are inherently sensitive to physical scale and prone to labeling errors. A fixed pixel error corresponds to different physical distances at different magnifications, which can distort comparisons, whereas the probability of label errors increases with higher magnifications. If testing labels are not manually checked for precise correctness before evaluating the models, segmentation evaluations can be biased. This may favor lower-magnification evaluations, where high-magnification label errors are less influential after downsampling.

Finally, generalizability was not evaluated by any of the CPath studies. Whereas Neary et al.^20^ and Srisermphoak^34^ experimented with multiple datasets, training and evaluation were always handled on the same dataset. Furthermore, Srisermphoak et al. did not include stain normalization methods in their study, which are fundamental when working with WSIs and could influence ML results. Whereas models trained with high magnification/color may perform better on their respective dataset, generalizability of these models could differ significantly and should be evaluated as well.

## Conclusion

Despite routine use of maximum magnification and full color information inputs in CPath, direct empirical evidence supporting these defaults is limited. The available CPath studies and evidence from other CV domains indicate that substantial resolution reduction can preserve performance for ML tasks, especially when models are trained at the target scale. Excessive reduction harms fine-grained discrimination. For color, the single available histology study suggests that advanced grayscale encodings can preserve diagnostically relevant information for CNN-based pipelines while simple grayscale transformation is insufficient, aligning well with research from other CV domains. Collectively, the evidence favors task-adaptive optimization rather than inherited assumptions. Engineering models specifically for grayscale images and training models directly at reduced magnification could prove beneficial for model performance.

Further research should aim to provide more evidence on the effect of color and magnification in CPath. In particular, research on grayscale images in combination with prior stain normalization and custom ML model architectures should be further explored. Furthermore, the generalizability of models with different magnifications and color information should be explored to realistically evaluate model performance.

## Declaration of competing interest

The authors declare that they have no known competing financial interests or personal relationships that could have appeared to influence the work reported in this article.
