# Supplementary material for: Benefits of high-magnification and color information in computational pathology—An exploratory review
Source: J Pathol Inform. 2026 Jul 30;22:100701. doi: 10.1016/j.jpi.2026.100701 (PMC13521022; doi:10.1016/j.jpi.2026.100701)
Supplement: Supplementary file 1 [file mmc1.pdf]

Table S1: Extended overview of studies addressing the effect of magnification or spatial resolution on deep learning model performance.

Study	Domain	ML-Task	Dataset	Magnification / Resolution	Model Type	Main Metrics	Main Finding
Neary-Zajiczek et al.[20]	CPath	Classification	WSI - BACH, BreaKHis, Patch-Camelyon	4x, 10x, 20x, 40x	Different DL ensembles	Accuracy	Retrained models maintained high classification performance under substantial simulated resolution degradation.
Wiebe et al. [21]	CPath	Classification	WSI - TCGA lung cancer	4, 8, 16, 32, 64, and 128 µm/px	Inception-v3	AuROC	Robust classification was retained at 4–16 µm/px; subtype discrimination degraded at lower resolutions.
Peng et al. [22]	CPath	Object Detection	WSI - in-house	4x, 8x, 12x, 16x, 40x	Different CNNs	Mean average precision	Downsampling provides a trade-off between accuracy and computational efficiency.
Ardohain and Fei [23]	GSI	Segmentation	3DEP, NLCD, WorldView-2, Sentinel-2, Landsat-8	1.5, 1.85, 10.0, and 30.0 m/px	U-Net, DeepLab v3	F1-Score, mean IoU	Higher-resolution data improved performance, but gains were often modest.
Yang and Hu [24]	GSI	Classification	WHU-HI	0.04 up to 5.0 m/px	1D-CNN, 3D-CNN, SSRN	Accuracy	Intermediate spatial resolutions preserved classification performance in several settings.
Sabottke et al. [25]	MI	Classification	NIH chest X-ray	32, 64, 128, 224, 256, 320, 448, 512, and 600 px square	ResNet34, DenseNet121	AuROC	Performance improved up to moderate input resolutions and then plateaued.
Rajaraman et al. [26]	MI	Segmentation	Shenzhen CXR	32, 64, 128, 256, 512, 768, and 1024 px square	Inception-V3 UNet	IoU, Dice	Segmentation performance peaked at 256×256 pixels in the studied setting.
Hirahara et al. [27]	MI	Classification	NIH chest X-ray	32, 64, 160, 192, 224, 256, 320, 1024 px square	CNN	Accuracy	Best classification performance at 64 x 64 px resolution.
Wollek et al. [28]	MI	Classification, Object Detection	NIH chest X-ray	64, 128, 256, 512, 1024 px square	DenseNet-121	AuROC, IoU	Best classification performance at highest resolution. However marginal gains between 256 and 1024 px. Mixed results for object detection.

Abbreviations: **CPath** Computational Pathology, **GSI** Geospatial Imaging, **MI** Medical Imaging, **WSI** Whole-Slide Image, **DL** Deep Learning, **AuROC** Area under the Receiver Operating Characteristic curve, **IoU** Intersection over Union.

Table S2: Extended overview of studies addressing the effect of color information on deep learning model performance.

Study	Domain	ML-Task	Dataset	Color Conditions Tested	Model Type	Main Metrics	Main Finding
Srisermphoak et al. [34]	CPath	Classification, Segmentation	WSI - PANDA, OES, CAMELYON16	Grayscale: Luminance and ACSRM	Segmentation: UNet, ResNet50-UNet VGG19-UNet, SwinUNet, and MISSFormer; Classification: ResNet50, VGG19, and SwinTransformer	F1-Score, IoU	Advanced grayscale conversion preserved or slightly improved CNN-based performance in several settings while reducing file size.
Dias et al. [35]	GSI	Segmentation	ISPRS Vaihingen, ISPRS Potsdam, INRIA	Grayscale	W-Net, U-Net	F1-Score	RGB provided a consistent advantage, while grayscale remained competitive.
Yang and Hu [24]	GSI	Classification	WHU-HI	Reduction of hyperspectral bands	1D-CNN, 3D-CNN, SSRN	Accuracy	Using all available spectral bands was not beneficial for all models; selected reductions preserved performance.
Pei et al. [36]	MGI	Classification, Regression	micrographs of Mg-Li based alloys, in-house	Grayscale	VGG16	Accuracy, R^2	Grayscale inputs performed competitively; preprocessing choices had strong task-dependent effects.
Buhrmester et al.[37]	CV	Classification	PersonFinder, FlickrScene, CIFAR-10, CIFAR-100	Grayscale, different color spaces	CNN	Accuracy / error rate	Color importance was dataset- and class-dependent; grayscale worked well for shape- or texture-driven tasks.
De and Pedersen [38]	CV	Classification	ImageNet-COLORDISTORT	Different color spaces and distortions	DenseNet, ResNet, VGG19, GoogleNet, MobileNet, AlexNet	Accuracy	Removing or distorting color consistently reduced classification performance across architectures.
De [39]	CV	Segmentation	ADE20K	Different color spaces and distortions	FCN, PSP, UPerNet, Segmenter	Accuracy	Color removal caused consistent segmentation performance drops, although transformer-based models were more robust.
Bui et al. [40]	CV	Classification	RGB-D	Grayscale	CNN-RNN with k-means filterbank features	Accuracy	Grayscale models outperformed RGB models in the studied setup and reduced computation time.
Bhatta et al. [41, 42]	CV	Classification	WebFace4M, MS1MV2, Glint360K, WebFace12M	Grayscale	ResNet	Accuracy	No significant differences between the two approaches. Grayscale images allowed for larger training datasets in resource-constrained settings.
Al-Mohana et al. [43]	CV	Classification	Malaria blood smear images - in-house	Grayscale	CNN	Accuracy	RGB achieved higher accuracy than grayscale, but grayscale still produced high classification performance.

Abbreviations: **CPath** Computational Pathology, **GSI** Geospatial Imaging, **MGI** Micrograph Imaging, **CV** General Computer Vision, **WSI** Whole-Slide Image, **IoU** Intersection over Union.
